# Prolactin May Not Play a Role in Primary Antiphospholipid (Hughes') Syndrome

**DOI:** 10.1155/2011/248243

**Published:** 2011-04-07

**Authors:** Manoel Tavares Neves Junior, Carlos Ewerton Maia Rodrigues, Jozelio Freire de Carvalho

**Affiliations:** Disciplina de Reumatologia, Faculdade de Medicina, Universidade de Sao Paulo, Av. Dr. Arnaldo, No. 455, 3 Andar, Sala 3190, 01246-903 Sao Paulo, SP, Brazil

## Abstract

The relationship between prolactin (PRL) and the immune system has been demonstrated in the last two decades and has opened new windows in the field of immunoendocrinology. However, there are scarce reports about PRL in primary antiphospholipid syndrome (pAPS). The objective of this study was to evaluate PRL levels in patients with pAPS compared to healthy controls and to investigate their possible clinical associations. Fifty-five pAPS patients according to Sapporo criteria were age- and sex-matched with 41 healthy subjects. Individuals with secondary causes of hyperprolactinemia (HPRL) were excluded; demographic, biometric, and clinical data, PRL levels, antiphospholipid antibodies, inflammatory markers, and other routine laboratory findings were analyzed. PRL levels were similar between pAPS and healthy controls (8.94 ± 7.02 versus 8.71 ± 6.73 ng/mL, *P* = .876). Nine percent of the pAPS patients and 12.1% of the control subjects presented HPRL (*P* = .740). Comparison between the pAPS patients with hyper- and normoprolactinemia revealed no significant differences related to anthropometrics, clinical manifestations, medications, smoking, and antiphospholipid antibodies (*P* > .05). This study showed that HPRL does not seem to play a role in clinical manifestations of the pAPS, differently from other autoimmune rheumatic diseases.

## 1. Introduction

Prolactin (PRL) is a peptide hormone secreted from the anterior pituitary gland and regulated by tonic inhibition of the hypothalamus via dopamine [1]. It is secreted not only by the anterior pituitary gland, but also by many extrapituitary sites, including immune cells [2]. Pituitary secretion of PRL is stimulated by suckling and stress [2]. The relationship between PRL and the immune system has been demonstrated in the last two decades and has opened new windows in the field of immunoendocrinology [2]. PRL has multiple immunostimulatory effects and promotes autoimmunity. It increases the synthesis of IFN-gamma and IL-2 by Th1 lymphocytes [3]. Moreover, PRL activates Th2 lymphocytes with autoantibody production [3] and it has a role in reproduction, calcium metabolism, osmoregulation, and behavior [4]. 

Hyperprolactinemia (HPRL) has been described in both non-organ-specific autoimmune diseases such as systemic lupus erythematosus (SLE), rheumatoid arthritis, systemic sclerosis, and psoriatic arthritis, as well as organ-specific autoimmune diseases such as celiac disease, type 1 diabetes mellitus, Addison's disease, and autoimmune thyroid diseases [2]. However, there are scarce reports in the literature regarding the significance of PRL in antiphospholipid syndrome (APS). Recently, a study evaluated the clinical significance of HPRL in APS and concluded that PRL indirectly may play a role in the pathogenesis of APS [5]. 

The aim of the present study was to evaluate the prevalence and clinical significance of PRL levels in pAPS patients and to compare them with healthy controls.

## 2. Methods

### 2.1. Patients

This comparative, descriptive, case-control study was conducted at the Rheumatology Division of the Hospital das Clinicas da Faculdade de Medicina da Universidade de Sao Paulo. 

All patients fulfilled the 1997 revised Sapporo criteria for the diagnosis of APS [6]. Anthropometric data, clinical manifestations, and laboratory results from 55 pAPS patients were collected from the patients' medical charts and compared with sex-matched healthy controls. Exclusion criteria were presence of other autoimmune diseases, such as SLE, use of drugs that are known to affect levels of PRL (i.e., psychotropic drugs, thyroid hormones, glucocorticoids, and estrogens or contraceptives), and patients with secondary causes of HPRL, such as primary hypothyroidism, end-stage renal disease, or prolactinomas.

Comparative analyses were carried out between sex, age, and disease duration. Anthropometric measurements including weight (kg), height (cm), and body mass index (weight/height^2^) were also performed. The following clinical parameters were evaluated: venous thrombosis (documented deep vein thrombosis and/or pulmonary embolism), arterial thrombosis (at least one of the following: documented peripheral arterial thrombosis, stroke, transient ischemic attacks, or acute myocardial infarction), livedo reticularis, thrombocytopenia, recurrent spontaneous abortions, and *in utero* fetal loss. In addition to the laboratory assessment of serum PRL, all sera of the patients were screened also for anticardiolipin antibodies, lupus anticoagulant, C-reactive protein (CRP), and erythrocyte sedimentation rate (ESR).

### 2.2. Antiphospholipid Antibodies

IgG and IgM anticardiolipin antibodies (ACLs) were estimated at least twice using an enzyme-linked immunosorbent assay (ELISA) as previously described [7]. There was an interval of 12 weeks between each measurement. Briefly, 50 *μ*L of cardiolipin (Sigma, St Louis, MO, USA) dissolved at 50 *μ*g/mL in ethanol was used to sensitize polystyrene microtiter plates that were left to dry overnight at 4°C. Nonspecific binding sites were blocked using 30% heat-inactivated fetal calf serum in PBS (FCS/PBS) for one hour. Fifty microliters of each serum sample diluted 1 : 50 in FCS/PBS was added in duplicate to the plates, followed by alkaline phosphatase-conjugated goat antihuman IgG (Sigma, St Louis, MO, USA). The manufacturer's cutoff values were used. For the diagnosis of the syndrome, values above 20 GPL or MPL were considered positive according to the criteria established by Sapporo [6]. Lupus anticoagulant was measured according to international guidelines using activated partial thromboplastin time (APTT-Diagnostica Stago, France) and diluted Russell's viper venom time (dRVVT-Trinity Biotech, Wiclow, Ireland) [8].

### 2.3. Inflammatory Markers

CRP was detected by nephelometry and ESR by modified Westergreen.

### 2.4. PRL Measurement

They were measured by fluoroimmunometric assay (*AutoDELFIA Prolactin, PerkinElmer Life analytic Science, Turku, Finland*). Reference values for women are 2.0 to 15.0 ng/mL and for men are 2.0 a 10.0 ng/mL.

### 2.5. Statistical Analysis

Results are presented as mean and standard deviations or median (range) for continuous variables and as a number (%) for categorical variables. Data were compared by *t*-tests or by the Mann-Whitney test for continuous variables to evaluate differences among patients with pAPS and controls and among pAPS patients with and without HPRL. For categorical variables, differences were assessed by a chi-square test or Fisher's exact test. *P* values less than .05 were considered significant.

## 3. Results

Table 1 shows the demographic characteristics, anthropometric measures, PRL levels, and inflammatory markers of pAPS patients and controls. pAPS patients and controls were similar in regards to mean age, female gender, and Caucasian race. Patients had a mean disease duration of 93.13 ± 61.96 months. 

pAPS patients had a higher weight (74.54 ± 19.99 versus 63.45 ± 8.68 kg, *P* = .0009) and BMI (29.2 ± 7.32 versus 28.9 ± 7.85 cm, *P* = .0065) compared to controls. 

PRL levels were similar (Table 1) when comparing pAPS patients and healthy controls (8.94 ± 7.02 versus 8.71 ± 6.73 ng/mL, *P* = .876), as was the frequency of HPRL (9.1 versus 12.1%, *P* = .740). No subject had any sign or symptom related to HPRL, such as galactorrhea or menstrual disturbances.

Inflammatory parameters such as CRP (4.52 ± 4.67 versus 2.15 ± 2.60 mg/L, *P* = .0063) and ESR (13.81 ± 13.33 versus 5.92 ± 4.30 mm/1st hour, *P* = .0006) were higher in pAPS patients compared to controls (Table 1).

Comparison of the five hyperprolactinemic pAPS patients to those with normoprolactinemia revealed no significant differences related to demographics (age, female sex, and Caucasian race), anthropometrics (weight, height, and BMI), antiphospholipid antibodies, inflammatory markers (CRP and ESR), clinical manifestations (arterial, venous, obstetric events, stroke, deep venous thrombosis, pulmonary thromboembolism, angina, limb ischemia, myocardial infarct, and thrombocytopenia), drug use (warfarin, acetylsalicylic acid, chloroquine, and statin), current or previous smoking, and diagnosis of systemic arterial hypertension (*P* > .05) (Table 2).

## 4. Discussion

This study demonstrated that pAPS patients presented similar PRL levels compared to control (8.94 ± 7.02 ng/mL and 8.71 ± 6.73 ng/mL, respectively). However, among the patients (55) five (9.0%) showed hyperprolactinemia but an equal number was also found among the control group (41). Comparison between the hyperprolactinemic pAPS patients with normoprolactinemic patients revealed no significant clinical differences.

PRL enhances immunoglobulin production [9], which may contribute to increased autoreactivity. A variety of autoantibodies were observed in patients with HPRL, including antibodies to PRL, endothelial cells, cardiolipin, *β*2 glycoprotein I (*β*2 GPI), Ro and La [10]. Praprotnik et al. [5] observed that HPRL is not associated with increased thrombosis; however, HPRL was more common among patients who had lupus anticoagulant activity as well as with some of APS major manifestations such as obstetric complications and did not relate to thrombosis. On the other hand, our study did not find any statistically significant association between pAPS with HPRL and normoprolactinemia in relation the presence of lupus anticoagulant, anticardiolipin antibodies, and clinical manifestations as thrombotics ones. According to other studies, PRL levels do not interfere directly in the modulating plaquete function which suggests that the prothrombotic effect of this hormone may involve other cells types [11]. It is worth to mention that our population was possibly more homogenous (Table 1) than the one used by Praprotnik and that genetic background may influence the results.

Obstetric events are associated with HPRL and antiphospholipid antibodies [5, 12] with impaired endometrial differentiation before conception. This mechanism contributes to the high incidence of pregnancy complications in APS [12]. Expression of decidual markers such as PRL, tissue factor (TF), signal transducer, and activator of transcription 5 (Stat5), but not insulin-like growth factor-binding protein 1 (IGFBP-1), is significantly lower in samples obtained from aPL (+) patients when compared with aPL (−) group [12]. In other words, patients with recurrent pregnancy loss have distinct endometrial gene expression profiles depending on the presence or absence of circulating aPL antibodies. In our study, only (1/5) patients with HPRL had obstetric event (pregnancy loss in the first trimester), and no significant correlation was found when pAPS were compared with normoprolactinemia (*P* = .64). However, this finding may be explained by the relatively small number of patients in our study.

Another interesting point of this study is that a wide variety of APS clinical manifestations were analyzed, such as neurological, haematological, cardiac, pulmonary, and thrombotics ones, as well serological antibodies of this syndrome and anti-inflammmatory markers. In SLE patients, PRL may have an effect on autoantibody production. Thus, autoimmune rheumatic disorders can be accompanied by increased PRL levels. Previous studies have demonstrated that lupus is associated with this endocrine alteration. The prevalence of HPRL in patients with SLE in most of the series ranges from 13 to 35% [13–15]. Moreover, one study [16] evaluated whether antibodies to PRL play a role in SLE patients with associated HPRL. They studied 259 consecutive SLE patients and suggested that anti-PRL antibodies could be the cause of HPRL in a subset of SLE patients, especially those with particularly high serum PRL levels with a diagnosis of idiopathic HPRL. The patients with anti-PRL antibody had fewer clinical manifestations and less serological activity, indicating that the biological activity of PRL was attenuated by the autoantibody. A meta-analysis demonstrated a significant increase in PRL concentrations in SLE patients. PRL likely stimulates lupus disease activity. Serum PRL and disease activity have been positively associated, and abnormally high PRL levels during pregnancy in SLE also correlate with disease activity [17]. According to the aforementioned, the best evidence for the association between PRL and human disease exists for SLE [18]. Curiously, another study which evaluated the prevalence and clinical significance of HPRL in APS concluded that HPRL was negatively related to arthralgias, venous thrombosis, pulmonary microthrombosis, pulmonary hypertension in APS, and neurological manifestations in pAPS (*P* < .05) [5]. However, our study found no connection between clinical manifestations and HPRL.

Certain limitations of our study must be addressed. Serum PRL levels were only measured at a single time point in our study and, thus, discrete alterations in the 24-hour secretion pattern of the hormone may have been missed [19]. In addition, factors other than circadian rhythms, such as age [20], menstrual cycle [21], sleep [22], and acute stressors [23], could have influenced our results.

Finally, our study showed that HPRL does not seem to play a role in clinical manifestations of the primary antiphospholipid syndrome, making it different from other autoimmune rheumatic diseases as SLE.

## Figures and Tables

**Table 1:** Demographic characteristics, anthropometric measures, PRL levels, and inflammatory markers in patients with primary antiphospholipid syndrome (pAPS) and controls.

	pAPS	Controls	*P*
	*N* = 55	*N* = 41	
Age, years	42.03 ± 11.63	37.37 ± 11.81	.053
Female gender, *n* (%)	47 (85.5)	35 (85.4)	1.00
Caucasian race, *n* (%)	49 (89.1)	34 (82.9)	.548
Disease duration, months	93.13 ± 61.96	—	—
Weight, kg	74.54 ± 19.99	63.45 ± 8.68	.0009
Height, cm	155.53 ± 33.32	154.09 ± 34.94	.830
BMI, kg/cm^2^	29.2 ± 7.32	28.9 ± 7.85	.0065
Waist, cm	90.63 ± 16.74	81.83 ± 8.14	.0022
PRL levels, ng/mL	8.94 ± 7.02	8.71 ± 6.73	.876
HPRL, *n* (%)	5 (9.0)	5 (12.1)	.740
CRP, mg/L	4.52 ± 4.67	2.15 ± 2.60	.0063
ESR, mm/1st hour	13.81 ± 13.33	5.92 ± 4.30	.0006

Data are presented as means ± standard deviations or percentages; *t*-tests and chi-square tests were used.

**Table 2:** Comparison between primary antiphospholipid syndrome patients (pAPS) with hyperprolactin and normal PRL levels.

	pAPS with HPRL	pAPS with Normoprolactinemia	*P*
	*N* = 5	*N* = 50	
Age (years)	34	42	.053
Female gender, *n* (%)	5 (100)	42 (84)	1.00
Caucasian race, *n* (%)	3 (60)	46 (92)	.086
Disease duration, months	106 (27–189)	82 (1–224)	.279
Weight, kg	70 (57–90)	75 (47–156)	.237
Height, cm	161 (158–168)	162 (140–180)	.215
BMI, kg/cm^2^	27 (22.3–31.9)	26.75 (27.5–42.2)	.328
Waist, cm	81 (76–110)	91 (65–157)	.294
Sedentarism, *n* (%)	5 (100)	29 (58)	.144
Current smoking, *n* (%)	0	8 (16)	1.00
Previous smoking, *n* (%)	0	20 (40)	.147
Arterial event, *n* (%)	3 (60)	34 (68)	1.00
Venous event, *n* (%)	2 (40)	28 (56)	.649
Obstetric event, *n* (%)	1 (20)	21 (42)	.638
Stroke, *n* (%)	2 (40)	22 (44)	1.00
Sneddon syndrome, *n* (%)	0	11 (22)	.571
Limb ischemia, *n* (%)	2 (40)	6 (12)	.149
Systemic arterial hypertension, *n* (%)	1 (20)	23 (46)	.373
Acute Myocardial Infarction, *n* (%)	0	1 (2)	1.00
Angina, *n* (%)	0	7 (14)	1.00
Deep venous thrombosis, *n* (%)	2 (40)	23 (46)	1.00
Pulmonary thromboembolism, *n* (%)	1 (20)	9 (18)	1.00
Thrombocytopenia, *n* (%)	0	12 (24)	.574
CRP, mg/L	0.87 (0.64–19)	3.22 (0.3–17.1)	.494
ESR, mm/1st hour	8.1 (2–32)	9 (2–58)	.287
Lupus anticoagulant, *n* (%)	3 (60)	34 (68)	1.00
Anticardiolipin IgM, *n* (%)	1 (20)	9 (18)	1.00
Anticardiolipin IgG, *n* (%)	2 (40)	18 (36)	1.00
Warfarin use, *n* (%)	4 (80)	46 (92)	.391
Chloroquine use, *n* (%)	3 (60)	23 (46)	.659
Statin use, *n* (%)	1 (20)	17 (34)	1.00
Acetylsalicylic acid use, *n* (%)	2 (40)	29 (58)	.643

Data are presented as means (range or percentages); Mann-Whitney and Fischer tests were used. HPRL was defined as PRL > 10 ng/mL for men and >15 ng/mL for women.
